# Synthesis and Characterization of Diosgenin Encapsulated Poly-ε-Caprolactone-Pluronic Nanoparticles and Its Effect on Brain Cancer Cells

**DOI:** 10.3390/polym13081322

**Published:** 2021-04-18

**Authors:** Bijuli Rabha, Kaushik Kumar Bharadwaj, Debabrat Baishya, Tanmay Sarkar, Hisham Atan Edinur, Siddhartha Pati

**Affiliations:** 1 Department of Bioengineering and Technology, Gauhati University, Guwahati, Assam 781014, India; bijulipep@gmail.com (B.R.); kkbhrdwj01@gmail.com (K.K.B.); 2 Malda Polytechnic, West Bengal State Council of Technical Education, Govt. of West Bengal, Malda, West Bengal 732102, India; tanmays468@gmail.com; 3 Department of Food Technology and Biochemical Engineering, Jadavpur University, Kolkata, West Bengal 700032, India; 4 Health Campus, School of Health Sciences, Universiti Sains Malaysia, Kubang Kerian, Kelantan 16150, Malaysia; 5 Centre of Excellence, Khallikote University, Berhampur, Ganjam, Odisha 761008, India; 6 Research Divisions, Association for Biodiversity Conservation and Research, Balasore, Odisha 756001, India

**Keywords:** Diosgenin, nanoparticles, glioblastoma cell lines, encapsulation

## Abstract

Diosgenin encapsulated PCL-Pluronic nanoparticles (PCL-F68-D-NPs) were developed using the nanoprecipitation method to improve performance in brain cancer (glioblastoma) therapy. The nanoparticles were characterized by dynamic light scattering (DLS)/Zeta potential, Fourier-transform infrared (FTIR) spectra, X-ray diffraction (XRD), Field Emission Scanning Electron Microscopy (FESEM), and Transmission electron microscopy (TEM). The encapsulation efficiency, loading efficiency, and yield were calculated. The in vitro release rate was determined, and the kinetic model of diosgenin release was plotted and ascertained. The cytotoxicity was checked by MTT (3-[4,5-dimethylthiazol-2-yl]-2,5-diphenyl tetrazolium bromide)assay against U87-MG cells (glioblastoma cell lines). The obtained nanoparticles demonstrated good size distribution, stability, morphology, chemical, and mechanical properties. The nanoparticles also possessed high encapsulation efficiency, loading efficiency, and yield. The release rate of Diosgenin was shown in a sustained manner. The in vitro cytotoxicity of PCL-F68-D-NPs showed higher toxicity against U87-MG cells than free Diosgenin.

## 1. Introduction

Cancer is the most dangerous critical disease that affects people worldwide, with one in every six deaths due to cancer [1]. Various therapeutic approaches, such as surgery, radiotherapy, chemotherapy, laser-based therapy, combined therapy, etc., are currently under practice, with chemotherapy used in nearly 50% of human cancer treatments [2]. The chemotherapeutic regimens often exert various toxicities related to cardiological, neurological, bone marrow suppression, chronic liver damage, gastrointestinal lesions, and many other side effects [3]. Glioblastoma is one of the most aggressive tumors that possess the ability to invade nearby brain surroundings through the process of angiogenesis [4], and about 100,000 people are diagnosed with glioblastoma every year [5]. An effective treatment for brain glioblastoma is still under development. The average survival rate for individuals after diagnosis of glioblastoma is about 15 months [6]. Therefore, new therapeutic strategies for treating glioblastoma are in urgent need [7].

Natural phyto-components derived from medicinal plants exhibit pharmacological properties and play an important role in the treatment of human diseases, including cancer, cardiovascular disease, diabetes, obesity, and metabolic and neurological diseases [8]. Studies have shown that these components have enormous potential, including the potential to lessen cell proliferation, inhibit cell metastasis, and induce cell apoptosis [9]. For example, some plant-derived phytocompounds such as steroidal saponins and their derivatives have promising anticancer activities [10]. These include Diosgenin(D), a naturally occurring steroidal sapogenin found in many plant families such as Agavaceae, Dioscoreaceae, Liliaceae, and Solanaceae [11,12]. Diosgenin has been reported as an apoptosis inducer of different types of cancer by inhibiting various cell signaling pathways in cells [13]. The anticancer effect of Diosgenin has been demonstrated in various preclinical research on cancer cells such as lung, breast, leukemia, colon, prostate, hepatocellular carcinoma, and squamous cell carcinoma [10,14,15,16,17,18].

Diosgenin has low toxicity effects compared with other pharmaceutical drugs, but its therapeutic efficacy and applications have been limited by poor water solubility (0.02 mg/L) and permeability, as well as low stability and bioavailability [14,19,20,21]. So, an effective approach is required to enhance the potential value of Diosgenin, including its anticancer efficacy. Nanomedicine is one of the best therapeutic approaches for drug delivery applications to enhance cytotoxicity in tumor tissues, reduce side effects on normal cells, reduce systemic toxicity, increase drug solubility, and increase maximum tolerated dose [22,23,24]. Several nanoparticle formulations have been recruited for drug delivery applications [25,26,27]. Among these, biodegradable polymer nanoparticles are most frequently utilized as drug delivery carriers due to their tunable properties, such as their small size, high loading and encapsulation efficiency, sustainable controlled release, and surface modification [28]. Encapsulation of drugs into polymer nanoparticles enhances bioavailability, solubility, release, efficiency, specificity, and drug therapeutic index values [29,30,31]. Several therapeutic drugs, as well as potential bioactive components, have been successfully encapsulated into polymer nanoparticles, which has improved their therapeutic efficacy, bioavailability, and controlled drug delivery [32,33]. Interestingly, polycaprolactone (PCL) nanoparticles have attained immense attention because their biocompatibility characteristics and usage has been approved by the Food and Drug Administration (FDA) [34]. PCL is a hydrophobic polymer formed by ring opening polymerization of ε-caprolactone into PCL [35]. In a previous study, the potential of PCL NPs as a drug delivery system was evaluated by testing the interactions with RAW 264.7 mouse murine macrophage cell line. Pegylated and non-pegylated PCL nanoparticles were investigated for the toxicity, immunological impact, and potency of macrophage endocytosis [36]. In another study, PCL-based nanocarriers efficiently inhibited the MDA-MB-231 cell proliferation, and researchers reported the enhanced survival rate of mice bearing B16 tumors [36]. A gelatin-coated PCL nanoparticle for naringenin delivery was reported to save mesenchymal stem cells(MSCs) from Oxygen Glucose Deprivation (OGD)-induced inflammatory stress [37]. Moreover, modified docetaxel-loaded PEG/PCL nanoparticles reported to target U-87 MG glioblastoma cell line showed successful cellular internalization and spheroid uptake with enhanced tumor penetration [38].

The goal of this study was to explore the efficacy of the nanoprecipitation method in order to evaluate the anticancer effects of Diosgenin-loaded polycaprolactone(PCL) nanoparticles using Pluronic F68 as surfactant (PCL-F68-D-NPs) against U87-MG glioblastoma cell line, which was reported by us for the first time. Further, we compared our results with that of free Diosgenin. The as-synthesized nanoparticles were characterized for their morphological properties, physicochemical properties, Diosgenin loading and encapsulation efficiency, and in-vitro release kinetics. The cytotoxicity study exerted by nanoparticles was evaluated by MTT (3-[4,5-dimethylthiazol-2-yl]-2,5-diphenyl tetrazolium bromide)assay and cell morphology was visualized by inverted microscope. Furthermore, the live/dead cells were visualized by confocal laser scanning microscopy analysis.

## 2. Materials and Methods

### 2.1. Materials

Poly-ε-caprolactone (PCL) (C6H10O2)n (Mw: 45,000 g/mol; density: 1.14 g/cm^3^) and Diosgenin (Mw: 414.62 g/mol;density: 1.1 g/cm^3^) were purchased from Sigma Aldrich (3050 Spruce Street, Saint Louis, MO, USA).Pluronic F-68 (Mw: 8400 g/mol)was purchased from Himedia (Mumbai, India), and Acetone (Merck, GmBH Germany) and deionized water were procured from the laboratory for experiments. The purity of chemicals was 98.9%. Pluronic F-68 was used as a surfactant.

The glioblastoma (U87- MG) cell lines were obtained from the National Centre for Cell Science (NCCS), Pune, India. These cell lines were cultured in Dulbecco’s Modified Eagle’s medium (DMEM) containing NaHCO_3_ (3.7 g/L) and D-glucose (4.5 g/L), supplemented with 10% FBS and 1% penicillin/streptomycin at 37 °C in 5% CO_2_.

### 2.2. Nanoparticle Preparation

Encapsulated Diosgenin PCL-Pluronic nanoparticles (PCL-F68-D-NPs) were prepared by the nanoprecipitation method. First, PCL (35 mg) and Diosgenin (2 mg) were dissolved/dispersed in 3 mL acetone by sonication for 1 min. The mixture was added drop-wise to 20 mL Pluronic F-68 (1%, *w/v*) under gentle magnetic stirring at 25 °C for 2 h. Nanoparticles were centrifuged at 12,000 rpm for 20 min and washed thrice with distilled water to remove un-encapsulated Diosgenin. The blank PCL-F68-NPs nanoparticles without Diosgenin were also prepared in the same manner. The nanoparticles were redispersed in distilled water and lyophilized. The lyophilized nanoparticles were stored at 4 °C until use.

### 2.3. DLS, PDI, and Zeta Potential

The quantification of size by dynamic light scattering (DLS), polydispersity index (PDI), and ζ-potential was performed using a Zetasizer Nano ZS90, (Malvern, UK). The blank PCL-F68-NPs, Diosgenin-loaded nanoparticles (PCL-F68-D-NPs) samples were diluted with distilled water and sonicated for several minutes before measurement. Data were obtained by the mean of 3 measurements [39].Particle size distribution can be determined by measuring the random Brownian motion changes in the intensity of light scattered from nanoparticles’ suspension or solution, whereas Zeta potential measures the surface charge of nanoparticles. The PDI is a parameter that determines the spread of the particle size distribution. The PDI can be calculated from the following:

PDI = (σ/d)^2^, where PDI = square of the standard deviation divided by the mean particle diameter.

### 2.4. FTIR

The Fourier-transform infrared (FTIR) spectra of PCL, Diosgenin, and Diosgenin-loaded nanoparticles (PCL-F68-D-NPs) were analyzed using a FTIR spectrophotometer (Perkin Elmer spectrum 100 FTIR, 710 Bridgeport, CT, USA). The samples were mixed with KBr in the ratio of 1:100 (*w/w*), i.e., 1 mg samples per 100 mg of KBr to prepare the pellets. The spectrum was scanned with wave numbers ranging from 4000 cm^−1^ and 400 cm^−1^ at a resolution of 4 cm^−1^ [40].

### 2.5. XRD

The diffraction patterns of PCL, Diosgenin, and Diosgenin-loaded nanoparticles (PCL-F68-D-NPs) were determined using a Phillips X’Pert Pro Powder X-ray diffractometer (XRD) (PANalytical, Almelo, The Netherlands). The samples were scanned from 2θ = 5° to 60° using a beam of Cu Kα radiation of wavelength, λ = 0.1542 nm, operated at 45 kV, 40 mA [41,42].

### 2.6. FESEM

A small amount (1 mg/mL) of the Diosgenin-loaded nanoparticles (PCL-F68-D-NPs) sample was drop-cast on a clean coverslip and dried. The samples were then sputtered with gold and observed under a field emission scanning electron microscope (FESEM) (Sigma Zeiss, Germany).

### 2.7. TEM

One drop (10 µL) of 1 mg/mL freshly prepared nanoparticles solution was placed on a copper grid and dried for 1 h. Thedried grid was examined by transmission electron microscope (TEM) JEOL 2100 Electron Microscope (JEOL, Peabody, MA, USA), operating at 120 kV acceleration.

### 2.8. Diosgenin Loading, Encapsulation Efficiency, and Yield of Production

The Diosgenin loading (%DL), encapsulation efficiency (%EE), and yield of production (%YP) of Diosgenin-loaded nanoparticles (PCL-F68-D-NPs) were determined spectrophotometrically. First, 1 mg was dissolved in 1.0 mL of acetone and incubated for 2 h for the complete extraction of Diosgenin from the nanoparticles. The solution was filtered through a 0.2 μm syringe filter. Then, perchloric acid was added to develop color and estimated by UV/Vis spectrometer (Synergy H1 microplate reader, BioTek Instruments, Inc., Winooski, VT, USA) at 489 nm. The amount of Diosgenin was calculated by referring to the standard curve of Diosgenin with various concentrations (1.0−1000 μg/mL). All the experiments were repeated in triplicate.

(1) $$\mathrm{DL}\%=\frac{Weight\;of\;diosgenin\;in\;NPs\;}{Weight\;of\;NPs}\times 100\;\%$$

The %EE was determined indirectly during nanoparticles synthesis. The synthesized nanoparticles were centrifuged at 12,000 rpm for 20 min at 4 °C. The supernatant containing the free Diosgenin was determined by UV/Vis spectroscopy. The amount of Diosgenin was estimated through the standard curve of Diosgenin at 489 nm.

(2) $$\mathrm{EE}\%=\frac{Amount\;of\;diosgenin\;encapsulated}{Total\;Diosgenin\;used\;during\;preparation}\times 100\;\%$$

(3) $$\mathrm{YP}\%=\frac{Weight\;of\;NPs\;recovered}{Weight\;of\;polymer\;and\;diosgenin\;fed\;initially}\times 100\;\%$$

### 2.9. In Vitro Drug Release Assay and Release Kinetics 

The release rate of Diosgenin from nanoparticles was determined by spectroscopic absorption analysis.

Briefly, 1 mg of Diosgenin-loaded nanoparticles (PCL-F68-D-NPs) was dispersed in 10 mL of phosphate-buffered saline (PBS) with pH 6.5 and 7.4 and was allowed to incubate at 37 °C. A pH level of 7.4 is a physiological condition that allows us to study in vivo performance. On the other hand, pH 6.5 represents the acidic tumor condition. Cancer tumors have been reported to maintain an acidic pH [43]. At different time intervals (1 h, 2 h, 3 h, 4 h, 5 h, 10 h, 24 h, and 48 h), an aliquot of 1.0 mL of sample was withdrawn and replaced with fresh PBS. The samples were centrifuged at 12,000 rpm for 20 min. Perchloric acid was added for color development to the supernatant containing Diosgenin and analyzed using a spectrophotometer (Synergy H1 microplate reader, BioTek Instruments, Inc., Winooski, VT, USA) at 489 nm. The Diosgenin concentration was calculated against an appropriate Diosgenin calibration curve. To determine the Diosgenin release kinetics, the release data were plotted on various kinetic models such as the zero-order model (cumulative percentage of drug released versus time), first-order model (log cumulative percentage of drug remaining versus time), Higuchi model (cumulative percentage of drug released versus square root of time), and Korsmeyer–Peppas model (log cumulative percentage of drug released versus log time).The model that fit best was used to represent the pattern of Diosgenin release from nanoparticles.

The mathematical equations for various release kinetic model are:

Zero-order model: F = K × t

First-order model: ln(1 − F) = −K × t

Higuchi model: F = K × t ^½^

Korsmeyer–Peppasmodel: F = Kt^n^

where F represents the cumulative percentage of drug released in time t, K is the rate constant, and n is drug release exponent.

When n ≤ 0.45, it indicates Fickian diffusion.

When n = 0.45 to 0.89, it indicates a non-Fickian type of release. A non-Fickian release determines both diffusion and erosion mechanisms.

When n = 0.89, it indicates Case II Transport by erosion.

### 2.10. In Vitro Cytotoxicity Study of Diosgenin-Loaded Nanoparticles

In vitro cytotoxicity study of free Diosgenin and PCL-F68-D-NPs at various concentrations (1–100 µM i.e., 1 µM, 5 µM, 10 µM, 20 µM, 40 µM, 60 µM, 80 µM, and 100 µM Diosgenin equivalent concentrations, respectively) was studied using MTT assay. Human glioblastoma cells (U87-MG) were purchased from the NCCS, Pune, India, and grown on DMEM media. Around 1 × 10^4^ cells were seeded in 96-well plates and incubated at 37 °C, 5% CO_2_ overnight. The cells were then treated with different concentrations of free Diosgenin and PCL-F68-D-NPs and incubated for 24 h. A working solution of 0.5 mg/mL of MTT was then added to each well and incubated at 37 °C for 4 h in the dark. After incubation, MTT was removed and DMSO (100 μL) was added to each well. The absorbance was read at 570 nm using a Synergy H1 microplate reader. The percentage of cell viability and IC_50_ was determined using the following equation:

(4) $$\mathrm{Cell}\;\mathrm{Viability}\;\%=\frac{Absorbance\;of\;sample}{Absorbance\;of\;control}\times 100\;\%$$

### 2.11. Cell Morphology of Diosgenin-Loaded Nanoparticles

Morphology of the cells was observed under an inverted microscope. Briefly, U87-MG cells were seeded at 1 × 10^4^ cells per well in 96 well plates. After 24 h, cells were incubated with or without a concentration of 1 mg/mL of Diosgenin, PCL-F68-D-NPs and kept for 24 h. The comparative effect of untreated, free Diosgenin and PCL-F68-D-NPs on U87-MG cell morphology was evaluated.

### 2.12. Live/Dead Assay

A live/dead assay was conducted for qualitative assessment of cell viability after treatment of nanoparticles.U87-MG cells at a density of 2 × 10^4^ cells were seeded in 96-well plates for 24 h. After that, cells were treated with or without PCL-F68-D-NPs and incubated for 24 h. After incubation, the cells were washed with PBS and stained with Thiazole orange (TO) and Propidium iodide (PI). The plates were then incubated for 30 min and observed using a confocal laser scanning microscope (CLSM).

### 2.13. Statistical Analysis

All data were expressed as the standard error of the mean (SEM) and assays were repeated thrice to ensure reproducibility. Statistical analysis was done through GraphPad prism software (version 8.01, GraphPad Software Inc., San Diego, CA, USA). The MTT data, i.e., the dose concentration and cell viability for Diosgenin as well as Diosgenin-loaded nanoparticles (PCL-F68-D-NPs),were analyzed by ANOVA. Analysis of variance (ANOVA) was performed followed by Tukey’s test for post-hoc analysis to compare the treated samples with the control groups. A *p*-value < 0.05 was considered statistically significant.

## 3. Results and Discussion

This study explored the therapeutic potential of Diosgenin-loaded nanoparticles (PCL-F68-D-NPs) in the glioblastoma cell line. The nanoparticles were synthesized by the nanoprecipitation method. The method was optimized to synthesize the nanoscale-level particles by changing their organic phase (i.e., acetone) and using different concentrations of pluronic F-68 (i.e., 1% final concentration, *w/v*). The method for the formulation of nanoparticles is dependent on several criteria, including the polymer, surfactant and drug concentration, the molecular weight of the polymer, aqueous and organic phase, stirring speed and time, temperature, etc., that control the size of the synthesized nanoparticles [44]. The synthesized nanoparticles were finally characterized by various techniques, such as DLS, PDI, Zeta potential, FTIR, XRD, FESEM, and TEM, and we further analyzed their efficacy in U87-MG cells.

### 3.1. DLS, PDI, and Zeta Potential

The prepared nanoparticles were subjected to DLS and Zeta potential measurements for the analysis of size, polydispersity index, and surface charge of the nanoparticles. Size of the blank nanoparticles (PCL-F68-NPs) and Diosgenin-loaded nanoparticles (PCL-F68-D-NPs) were accounted to be 193.7 nm and 245 nm with polydispersity indices (PDI) of 0.463 and 0.367 respectively, with an equivalent size distribution (Supplementary Figure S1).The noticeable increase in size of the PCL-F68-D-NPs as compared to blank PCL-F68-NPs was due to the loading of Diosgenin. The nanoparticle size plays an important role in the delivery of therapeutic drugs or components [44]. The cellular uptake of nanoparticles is also dependent on the nanoparticle size [45,46]. Nanoparticles that are smaller in size, i.e., less than 300 nm, can be easily transported throughout human body [44]. Moreover, the Zeta potential of the Diosgenin-loaded nanoparticles (PCL-F68-D-NPs) was found to be −22.3 mV and −11.5 mV (Table 1). This characterizes stable colloidal dispersion of the nanoparticles with less tendency for aggregation [47]. The PCL does not have a negative charge, but in water, the carboxylic groups may be ionized due to the formation of anionic carboxylate ions. The PCL showed negative Zeta potential [48]. Similar reports were observed when celastrol was encapsulated in PCL nanoparticles [39]. The decrease in the Zeta potential value, i.e., from relatively more negative to less negative Zeta potential, may have been due to the reaction of Diosgenin and PCL in water-based media at the interface. An earlier report suggested that the hydrophobic surfaces with and without functional groups attain surface charge due to the substitution of the adsorbed water molecules with ions (OH^−^, H3O^+^) and acid–base reactions between the liquid medium, respectively [49].

### 3.2. FTIR

**Figure 1:**
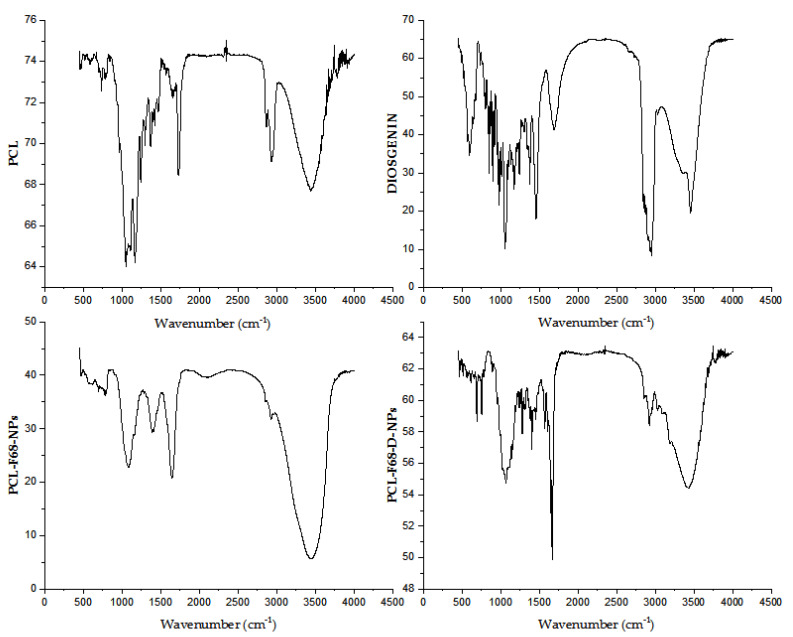
Fourier-transform infrared (FTIR) spectra of polycaprolactone (PCL), Diosgenin, blank nanoparticles (PCL-F68-NPs), and Diosgenin-loaded nanoparticles (PCL-F68-D-NPs).

The Fourier-transform infrared (FTIR) spectra for pure Diosgenin, blank nanoparticles (PCL-F68 NPs), and Diosgenin-loaded nanoparticles (PCL-F68-D-NPs) are illustrated in Figure 1. The Diosgenin spectrum showed bands at 3456 cm^−1^, 2946 cm^−1^, and 1447 cm^−1^ and strong characteristic peaks at 1163 cm^−1^ and 1058 cm^−1^. A band at 3456 cm^−1^ corresponds to -OH stretching, and the bands at 2946 cm^−1^ and 1447 cm^−1^ corresponding to CH_2_ stretching and scissoring vibration, respectively. Strong characteristic peaks at 1163 cm^−1^ and 1058 cm^−1^ can be attributed to -C-O stretching, and the band at 898 cm^−1^ is assigned anCH_2_ twist. This spectrum of Diosgenin matched with previous results as reported earlier in literature [50].

The blank PCL-F68 NPs showed characteristic peaks of PCL at 2923 cm^−1^ and 2856 cm^−1^, respectively. Other peaks at 1647 cm^−1^, 1395 cm^−1^, and 1076 cm^−1^ were observed. The PCL-F68-NPs showed a band with asymmetric and symmetric stretching of CH2 at 2923 cm^−1^ and 2856 cm^−1^, respectively. Moreover, other peaks were observed, such as carbonyl-stretching at 1647 cm^−1^, whereas the CH_2_ bending and O-H bending at 1395 cm^−1^, and C–O stretching at 1076 cm^−1^ were observed instead of its normal stretching at 1720 cm^−1^. This shifting of carbonyl stretching from 1720 cm^−1^ for PCL to 1647 cm^−1^ in PCL-F68-NPs might have been due to an interaction with water accumulated during the formulation drying step.

The loaded PCL- F68- D NPs showed all the peaks of PCL-F68 NPs with new IR absorption at 1672–1286 cm^−1^ and 3183–3024 cm^−1^ that might be attributed to an interaction between Diosgenin and the polymer. A band at 1647 cm^−1^ related to PCL-F68 NPs was shifted to 1672–1286 cm^−1^ in PCL-F68-D-NPs, whereas the Diosgenin band at 3456 cm^−1^ was shifted to 3183–3024 cm^−1^ in PCL-F68-D-NPs. This report supported the successful encapsulation of Diosgenin into the formulated nanoparticles.

### 3.3. X-ray Diffraction

**Figure 2:**
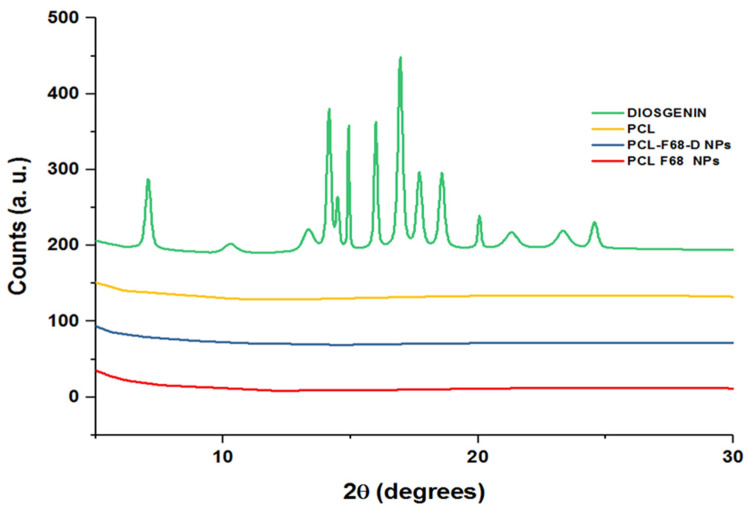
X-ray diffraction (XRD) spectra of PCL, Diosgenin, PCL-F68-NPs, and PCL-F68-D-NPs.

XRD pattern ascertains the crystal structure of compounds and determines various structural characteristics of the crystalline state. Figure 2 shows the X-ray diffraction (XRD) patterns of pure Diosgenin and Diosgenin-loaded nanoparticles (PCL-F68-D-NPs). Pure Diosgenin exhibited the typical crystalline peaks observed at 2θ = 6.96°, 10.42°, 11.49°, 13.37°, 14.04°, 14.86°, 16.01°, 17.00°, 17.82°, 18.64°, 20.13°, 21.27°, 23.42°, and 24.58°. However, the characteristic peaks related to the synthesized PCL-F68-D-NPs did not display any such crystalline peaks. The absence of any detectable crystalline peaks of Diosgenin in the PCL polymer nanoparticles can be attributed to an amorphous or disordered state and suggests its encapsulation into PCL-PE-N-NPs [51,52]. Moreover, the case of PCL polymer and blank PCL-F68 NPs revealed no detectable peaks as they share similar amorphous nature with PCL-F68-D-NPs.

### 3.4. FESEM

**Figure 3:**
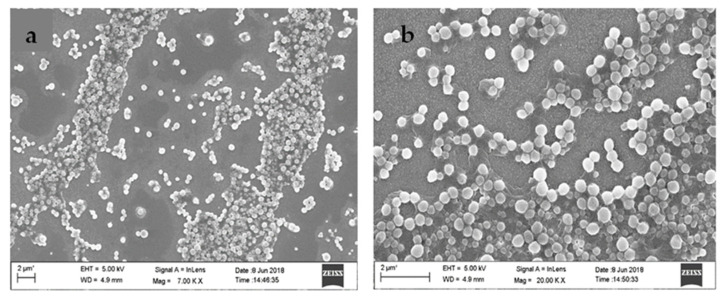
Field emission scanning electron microscope (FESEM) images (**a**,**b**) of Diosgenin-loaded nanoparticles (PCL-F68-D-NPs).

FESEM analysis was executed to study the surface morphology of synthesized nanoparticles. The FESEM images in Figure 3 revealed spherical morphology for Diosgenin-loaded nanoparticles (PCL-F68-D-NPs) with a smooth surface. The size distribution of FESEM analysis revealed nanoparticles of 90–100 nm which were almost uniform in nature. The differences in size obtained by DLS (>200 nm) was due to the dispersion in liquid medium, producing a hydrodynamic nature of the nanoparticles. During FESEM analysis, the observed smaller size of the nanoparticles was due to shrinkage in the coverslip in the dry state. This result is in agreement with earlier findings [53,54].

### 3.5. TEM

**Figure 4:**
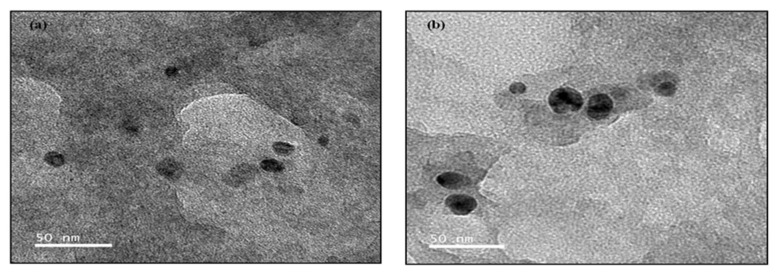
Transmission electron microscope (TEM) image (**a**,**b**) of Diosgenin-loaded nanoparticles (PCL-F68-D-NPs).

The morphological characterization of TEM results showed (Figure 4) that the Diosgenin-loaded nanoparticles (PCL-F68-D-NPs) were moderately spherical in nature. This finding is consistent with a previous study that showed the uniformly spherical shape, which is almost in agreement with the FESEM a result. Most of the synthesized nanoparticles were smaller than 100 nm in size. The size of the nanoparticles influences the drug release rate and cellular uptake as well as tissue uptake. It has been reported that the uptake efficiency of nanoparticles with sizes of 100 nm increases by 2.5–250 fold compared to micro-particles [45]. Smaller nanoparticles can be retained in the blood circulation for a longer period of time as compared to larger nanoparticles NPs [55].

### 3.6. Encapsulation Efficiency, Diosgenin Loading, Yield of Production

The drug loading and encapsulation efficiency of Diosgenin-loaded nanoparticles (PCL-F68-D-NPs) was evaluated using UV/Vis spectroscopy. A calibration curve was plotted to calculate the Diosgenin content in the nanoparticles and read at 489 nm. The encapsulation and loading efficiency of PCL-F68-D-NPs was found to be 80.8 ± 0.26% and 10.3 ± 0.31% respectively. The yield of the production of nanoparticles was 68.02 ± 0.1. The encapsulation, loading efficiency, and yield are recorded in Table 2. The solubility of drugs in polymer suspension also controls the loading and encapsulation efficiency [56]. This loading capacity of nanoparticles is directly associated with the structural, physical, and chemical properties that permit the void area to load drugs into it [56]. The drug loading and encapsulation efficiency analysis can be considered to enhance the therapeutic potential of drugs. Various factors such as drug-polymer-surfactant ratio and the temperature can influence the loading and encapsulation efficiency in the nanoparticles. The nature of the encapsulating molecules, nanoparticle molecules, the medium of synthesis, and the drug loading technique (incorporation of the drug during synthesis or absorption of the drug after synthesis) can determine the encapsulation efficiency [39,44]. Sanna et al. prepared celastrol-loaded PCL nanoparticles by the nanoprecipitation method. They reported an encapsulation efficiency of 65.2%, drug loading content of 3.26%, and yield of production of 50% with stable colloidal stability [39]. It was deduced that there was a high affinity between celastrol and the hydrophobic polymer. Earlier researchers also reported Diosgenin-conjugated poly (ε-caprolactone) as a co-delivery system for imatinib with %EE (60–85%) and %DL (10–15%), owing to good colloidal stability [57].

### 3.7. In Vitro Drug Release Studies

**Figure 5:**
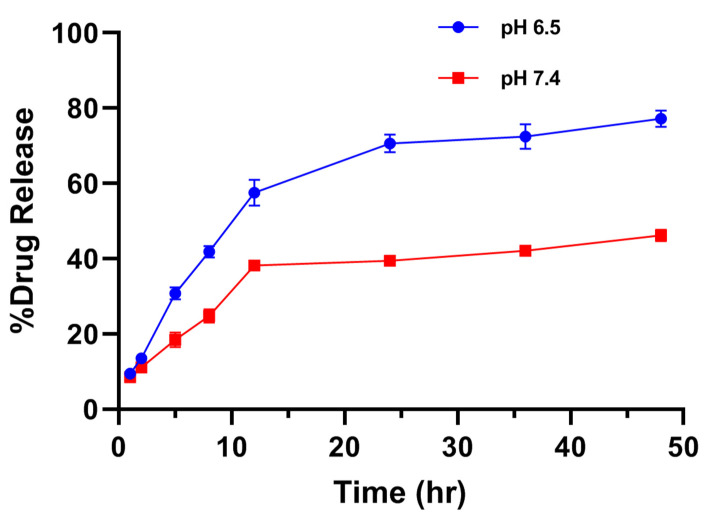
In vitro release profile of Diosgenin-loaded nanoparticles (PCL-F68-D-NPs) in phosphate-buffered saline (PBS) (pH 6.5 and pH 7.4) for 48 h. The data represent ± SEM of n = 3.

The release study of drugs is a very important parameter where the release profile of adsorbed or encapsulated drug has to be carefully examined. The in vitro drug release studies were performed in different simulated physiological pH levels (6.5 and 7.4 at 37 °C) of phosphate buffer saline for varying time intervals up to 48 h. The in vitro drug release was performed here by the in vitro dissolution method. The samples were evaluated for specific time intervals using UV/Vis spectroscopy at 489 nm. Absorbingly, the cumulative percentage release of free Diosgenin and Diosgenin-loaded nanoparticles (PCL-F68-D-NPs) remarkably varied. In vitro, drug release profile showed that the release of drug at pH 6.5 was more than at pH 7.4, as shown in Figure 5. It has been reported that tumor sites have lower pH than blood and normal cells or tissues [43]. Moreover, the results revealed a biphasic release pattern of Diosgenin from the polymeric nanoparticles. An initial burst release during the initial 8 h followed by sustained release was observed. The initial burst release of Diosgenin was due to the release from surface-adsorbed, non encapsulated or loosely bound Diosgenin diffusion from the polymer matrix during hydration of the nanoparticles [44,58]. The release rate was slower for the next 48 h, with up to 74% release for pH 6.5 and 44% for pH 7.4. The sustained release of Diosgenin transpired was due to the release of entrapped Diosgenin within polymer matrix when degrading, as well as hydrophobic interaction of polymer–Diosgenin within the nanoparticle. This prevented the faster diffusion of the Diosgenin from the nanoparticle and maintained the sustainable release for longer periods.

The drug release pattern was evaluated by plotting Diosgenin release data into various mathematical kinetic model equations, such as the zero-order, first-order, Higuchi, and Korsmeyer–Peppas kinetic models. The correlation coefficient (R^2^) from the linear regression curve was used to screen out the best-fitted release models. Based on the R^2^ values of different models, it was observed and concluded that the release of Diosgenin from the PCL nanoparticle matrix followed the Korsmeyer–Peppas model (R^2^ = 0.947 and 0.951), as shown in Table 3. These results suggest that release of Diosgenin is controlled by diffusion and dissolution from the polymeric matrix [59]. The dissolution media first diffused into the nanoparticle matrix through the pores, slowly dissolved the encapsulated Diosgenin, and released Diosgenin by the diffusion mechanism. Moreover, the release exponent value (n) for Diosgenin release was found to be 0.42 and 0.37 in pH 6.5 and pH 7.4, respectively. In this context, if the exponent value n ≤ 0.45, it specifies a Fickian diffusion type of release distinguished by a lower polymer relaxation time compared to the diffusion time of solvent, whereas if the n value lies between 0.45 to 0.89, it stipulates the non-Fickian type of drug release, i.e., an anomalous type of transport indicates the combined diffusion and erosion-controlled release mechanism. When n ≥ 0.89, the release is generally controlled by the erosion mechanism. It can be concluded that the smaller the n value, the greater release of drug ensued by diffusion, and the greater the n value, the greater release of drug ensued by erosion [44]. Since the release exponent n value in both conditions was less than 0.45, the result confirms the Fickian diffusion type of release of Diosgenin from the PCL-F68-D-NPs. Earlier, it was reported that the increase in drug-polymer interaction and molecular rearrangement of polymer chains can cause slower diffusion and release as the solvent diffuses into the nanoparticles, which is useful for targeted delivery [60,61].

### 3.8. In Vitro Cytotoxicity Assay

**Figure 6:**
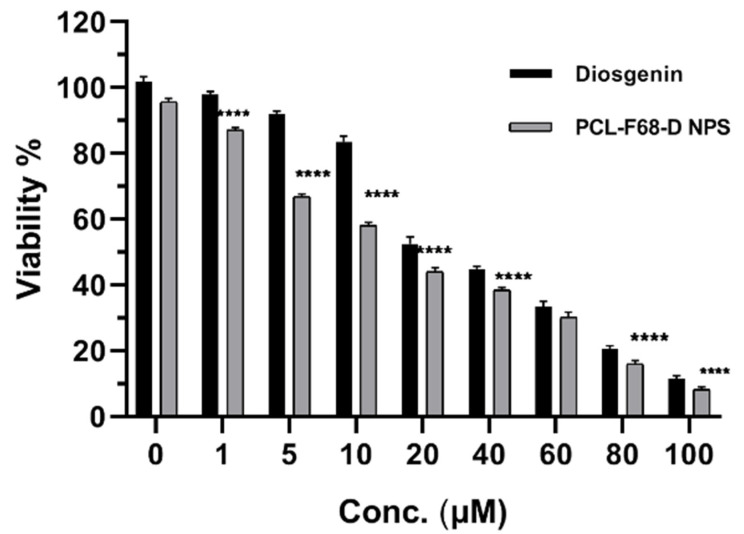
U87-MG glioblastoma cell cytotoxicity profile. MTT assay of Diosgenin and Diosgenin-loaded nanoparticles (PCL-F68-D-NPs) treated with varying concentrations (1 µM, 5 µM, 10 µM, 20 µM, 40 µM, 60 µM, 80 µM, and 100 µM) after 24 h incubation. Diosgenin 0 is untreated control; PCL-F68-D-NPs 0 is control group treated with blank PCL-F68-NPs. Graph data show **** *p* < 0.05). The data represent ± SEM of n = 3.

The anticancer activity of Diosgenin-loaded nanoparticles (PCL-F68-D-NPs) on glioblastoma cells, U87-MG, was studied. After incubation, cells were treated with different concentrations of Diosgenin and Diosgenin-loaded nanoparticles (PCL-F68-D-NPs) for 24 h. The concentration of Diosgenin ranged from 1–100 µM (1 µM, 5 µM, 10 µM, 20 µM, 40 µM, 60 µM, 80 µM, and 100 µM) for pure Diosgenin, as well as for PCL-F68-D-NPs calculated based on loading capacity, i.e., 10.3%. The MTT results in Figure 6 reveal that Diosgenin and PCL-F68-D-NPs both showed antiproliferative activity in a dose-dependent manner. A considerable decrease in the viable cells was observed at a higher concentration of 40 µM. Pure Diosgenin showed 44% viable cells while PCL-F68-D-NPs showed 37% viable cells, which clearly indicates that Diosgenin-loaded nanoparticles (PCL-F68-D-NPs) are more potent than pure Diosgenin against U87-MG cells. The effective IC_50_ values (IC_50_ value indicates the concentration of drug needed to inhibit the biological process by 50%) obtained for Diosgenin and PCL-F68-D-NPs were found to be at 28.89 ± 2.12 µM and 14.90 ± 0.304 µM, respectively, which was significantly lower than pure Diosgenin (*p* < 0.05).

Similar results were observed when Diosgenin-loaded polymer nanoparticles were introduced for in vitro cytotoxicity against A549 lung carcinoma cell cells. The analysis delineates that the IC50 values for free Diosgenin, PGMD 7:3, and PGMD 6:4 nanoparticles were 27.14 µM, 15.15 µM, and 13.91 µM, respectively, which were significantly lower (*p*< 0.05) when compared to the free Diosgenin [43]. Similarly, Diosgenin-loaded noisome showed better cytotoxicity against hepatocellular carcinoma and HepG2 cells as compared to free Diosgenin [14]. The mechanisms behind the better cytotoxicity of drug-loaded nanoparticles as compared to free drugs is adsorption on the cellular surface and the generation of concentration gradient, as well as cellular endocytosis [62].

### 3.9. Cell Morphology Assessment

**Figure 7:**
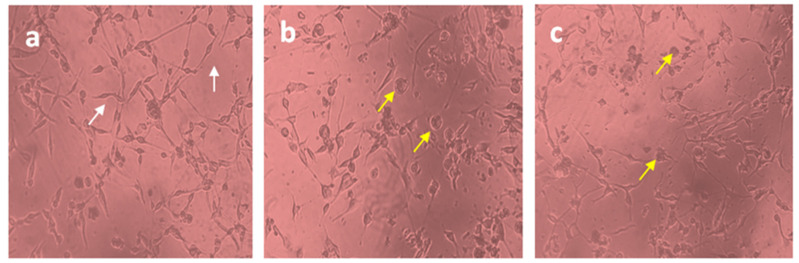
Cell morphology of U87-MG cells (10X). (**a**) Untreated U87-MG cells showed normal cellular structure (white arrows), (**b**)Diosgenin-treated cell, (**c**) PCL-F68-D-NPs-treated cell; cell rounding (yellow arrows).

The U87-MG glioblastoma cell line was treated with free Diosgenin and Diosgenin-loaded PCL-F68-D-NPs nanoparticles for 24 h. The morphological observations in untreated cells as control displayed distinct cell growth. A reduced number of cells in the population for Diosgenin PCL-F68-D-NPs nanoparticles treatment were noticed compared to untreated control cells, as expected. In addition, an elongated cell structure was observed in the control group, while a more shrunken and rounded cell structure was observed in both treated groups (Figure 7). The reduction of the cell population and cell roundness signifies the phenomenon of apoptosis.

### 3.10. Live/Dead Assay

**Figure 8:**
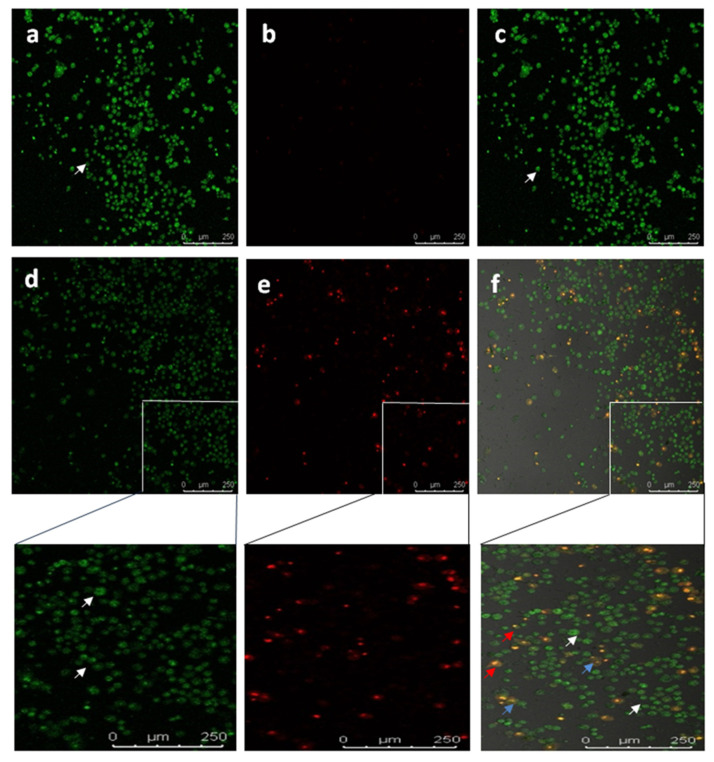
Confocal images showing live-dead (Thiazole orange/Propidium iodide) staining in U87-MG cells. Untreated U87-MG cells showed normal cellular structure (white arrows).Apoptotic cell (blue and red arrows) includes cell membrane blebbing and chromatin condensation, respectively. (**a**) TO, (**b**) PI, (**c**) overlay image of untreated; (**d**) TO, (**e**) PI, (**f**) overlay image treated with PCL-F68-D-NPs.

To study the apoptotic efficiency of Diosgenin-loaded nanoparticles (PCL-F68-D-NPs), live/dead assays were performed on U87-MG cells. The typical characteristics of an apoptotic cell encompass cell shrinkage, the formation of blebs, chromatin condensation, and nuclear disintegration. The nucleic acid-binding dyes thiazole orange (TO) and propidium iodide (PI) were used to stain and monitor live and dead cells under the microscope. TO is a permeant dye and thus stains all cells, live and dead. Meanwhile, PI is impermeable to live cells that have intact membranes and binds only to dead cells. The results clearly show that the live cells had green color fluorescence and dead cells had red color fluorescence. The overlayed image reveals a clear understanding of the apoptotic body with an orange nucleus (Figure 8) [63,64]. Similar morphological appearances were observed when Diosgenin was treated on U87-MG cell lines (result not shown). In the untreated control cells, large numbers of live cells were observed. On the other hand, as shown in Figure 8, PCL-F68-D-NPs-treated cells effectively underwent apoptosis. The cells clearly demonstrate nuclear condensation and deformed structure as compared to the control cells and other images [65,66].

## 4. Conclusions

In the present study, a potent anticancer compound Diosgenin was encapsulated into polymer nanoparticles by the nanoprecipitation method. The prepared nanoparticles obtained about <200 nm in size with a spherical and smooth surface with high encapsulation and Diosgenin loading efficiency. The in vitro release profile showed a biphasic pattern of release, i.e., an initial burst release followed by sustained release. Moreover, cell cytotoxicity assay proved that the Diosgenin-loaded nanoparticles (PCL-F68-D-NPs) killed and inhibited the effective proliferation of cancer cells in a dose-dependent manner. The cellular morphological change was evaluated after the administration of treatment with PCL-F68-D-NPs using an inverted microscope. The live/dead assay was performed for evaluation of apoptosis using TO/PI, which revealed that Diosgenin in nanoparticles induces apoptotic cell death in cancer cells. Therefore, these findings show that Diosgenin-loaded nanoparticles can be applied as a promising drug carrier for drug release and therapy in clinical diagnosis. Moreover, further studies are required to corroborate the experimental findings in the future.

## Figures and Tables

**Table 1:** Particle size, polydispersity index (PDI), and Zeta potential of formulated nanoparticles.

Batch	Particle Size (nm)	PDI	Zeta Potential (mV)
**PCL-F68 NPs**	193.7	0.463	−22.3
**PCL-F68 D NPs**	245.1	0.367	−11.5

**Table 2:** Diosgenin loading, encapsulation efficiency, and yield of production of formulated nanoparticles.

Polymer NPs	% DL	% EE	% YP
**PCL-F68-D NP**	10.3 ± 0.31%	80.8 ± 0.26%	68.02 ± 0.1

**Table 3:** Mathematical model for release kinetics dissolution data of PCL-F68-D-NPs.

Dissolution Condition	Zero Order (R^2^)	First Order (R^2^)	Higuchi Model (R^2^)	Korsmeyer–Peppas Model (R^2^)	Release Exponent Value (n)
**pH 6.5**	0.779	0.605	0.915	0.947	0.42
**pH 7.4**	0.776	0.645	0.894	0.951	0.37

## Data Availability

Provided in the above supplementary section.

## Supplementary Materials

The following are available online at https://www.mdpi.com/article/10.3390/polym13081322/s1, Supplementary Figure S1 (a) Particle size measurements with DLS and (b) zeta potential of synthesized Diosgenin loaded nanoparticles (PCL-F68-D-NPs).
