# Supplementary material for: Synthesis and Characterization of Diosgenin Encapsulated Poly-ε-Caprolactone-Pluronic Nanoparticles and Its Effect on Brain Cancer Cells
Source: Polymers (Basel). 2021 Apr 18;13(8):1322. doi: 10.3390/polym13081322 (PMC8073865; doi:10.3390/polym13081322)
Supplement: Supplementary file 1 [file polymers-13-01322-s001.zip › Supplementary Figure S1.pdf]

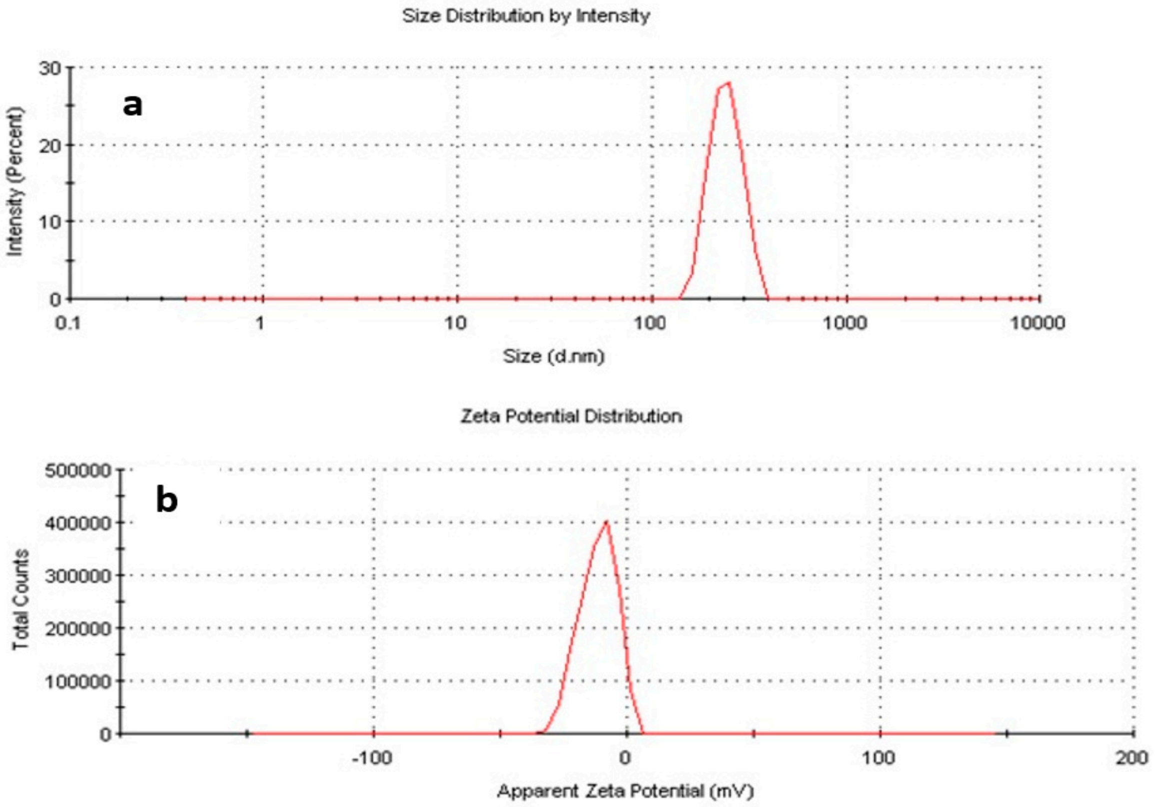

Supplementary Figure S1. (a) Particle size measurements with DLS and (b) zeta potential of synthesized Diosgenin loaded nanoparticles (PCL-F68-D-NPs).
